# Intestinal parasitic infections and malnutrition amongst first-cycle primary schoolchildren in Adama, Ethiopia

**DOI:** 10.4102/phcfm.v3i1.198

**Published:** 2011-05-12

**Authors:** Pawlos Reji, Getachew Belay, Berhanu Erko, Mengistu Legesse, Mulugeta Belay

**Affiliations:** 1 Oromia Health Bureau, Addis Ababa, Ethiopia; 2 Ethiopian Health and Nutrition Research Institute, Addis Ababa, Ethiopia; 3 Aklilu Lemma Institute of Pathobiology, Addis Ababa University, Addis Ababa, Ethiopia

## Abstract

**Background:**

A survey of intestinal parasitic infections and malnutrition in different regions or localities is a very important step in developing appropriate prevention and control strategies.

**Objectives:**

The objective of this study was to investigate the magnitude of intestinal parasitic infections and malnutrition amongst first-cycle primary schoolchildren in Adama town, Ethiopia.

**Method:**

A total of 358 children from four primary schools in Adama town were included for stool examination, weight for age, height for age, weight for height and socio-economic status of the family.

**Results:**

The result of stool examinations showed that 127 (35.5%) of the study subjects were infected by one or more parasite. The most frequent parasites were *Entamoeba histolytica/dispar* (12.6%) and *Hymenolopis nana* (8.9%). The rate of intestinal parasitic infection was not significantly associated with sex, age or socio-economic factors and nutrition (*P* > 0.05). The overall prevalence of malnutrition was 21.2%. Those children whose families had a monthly income of less than 200 ETB (Ethiopian birr) were highly affected by malnutrition (*P* < 0.05), but family education was not identified as a factor for malnutrition amongst schoolchildren.

**Conclusion:**

The prevalence of *E. histolytica/dispar* and *H. nana* could be of public health importance and calls for appropriate control strategies, and the high prevalence of malnutrition amongst children from poor families requires intervention.

## Introduction

Intestinal parasitic infections, especially helminths, are common health problems of children. Children at school age are at risk of developing clinical manifestation, because helminthic infections such as *Trichuris trichuria* and *Ascaris lumbricoides* reach maximum intensity at 5–10 years of age.^1^ It is estimated that for children 5–14 years of age in low-income countries, intestinal worms account for 12% of the total disease burden.^2^ This can be due to socio-economic and environmental factors.^3^ Studies in Africa have reported a high prevalence of intestinal parasitic infection amongst schoolchildren.^4, 5^ The magnitude of the problem varies amongst countries as well as in areas within countries.^4, 5^ Malnutrition is a common health problem of African schoolchildren due to intestinal parasitic infections and many other factors.^6^

The Ethiopian population falls mostly in the low socio-economic strata and health care coverage in the country is poor. The magnitude of infectious diseases, including intestinal parasites amongst children, is therefore expected to be high. Studies in many parts of the country have shown a high prevalence of intestinal parasitic infections amongst schoolchildren.^7, 8^ Children in Ethiopia are also highly affected by malnutrition due to multifactorial reasons.^9^

The prevalence of intestinal parasitic infections varies from place to place in Ethiopia, and there is therefore a need for local baseline data for better control and prevention strategies. The aim of this study was therefore to investigate the magnitude of intestinal parasitic infections and malnutrition amongst first-cycle primary schoolchildren in Adama town.

### Research significance

The prevalence of intestinal parasitic infections varies from place to place in Ethiopia and the magnitude of malnutrition is not known. Therefore, the research will be a baseline data for better control and prevention strategies.

## Ethical considerations

Before commencement of the study, the project was approved by the Ethical Clearance Committee of Aklilu Lemma Institute of Pathobiology and the Oromia Health Bureau. Consent was also obtained from children's parents and/or guardians, principals and class teachers after a verbal explanation of the procedures and purpose of the study. All children who tested positive for helminthiasis were treated with the appropriate drug.

## Methods

### Materials

Data collected included anthropometric measurements and laboratory examination stool specimens. Children's parents and/or guardians were interviewed to obtain socio-economic information such as income, educational status, ethnicity and religion.

#### Anthropometric measurement

Anthropometric measurements of height for age (HA), weight for age (WA) and weight for height (WH) were used to assess the nutritional status of the children. The weight and height of the children were measured to the nearest 0.1 units using standard measuring devices and methods. The ages of children were obtained from the school records. The Z-scores of HA, WA and WH were calculated using the EPINFO version 3.3 computer program according to World Health Organization (WHO) reference standards.^11^ Based on the WHO reference standards, those children with Z-scores below 2SD for HA, WA and WH were identified as stunted, underweight and wasted, respectively. For those children whose height was taller than or equal to 140 cm, a body mass index (BMI) with a cut-off value of 18.5 kg/m^2^ was used instead of Z-score of WH, and those children below this cut- off value were identified as wasted.^12^

#### Stool sample collection and examination

Stool samples were collected from 358 children and preserved in 10% formalin and then processed using the formol-ether concentration technique.^13^ The results were used to report the prevalence of intestinal parasitic infections. Kato-Katz thick smears were prepared from fresh stool samples for quantitative egg counts and the intensity of infection was reported based on the WHO criteria.^14^ For *Ascaris lumbricoides*, 1–4999 egg per gram (epg) was reported as light, 5000 to 49 999 epg as moderate and 50 000 epg and higher as heavy infection. For *Trichuris trichuria*, 1–999 epg were reported as light, 1 000–9 999 epg as moderate and 10 000 epg and higher as heavy infection. An ova count of 1–99 epg, 100–399 epg and 400 and higher epg was reported as light, moderate and heavy, respectively, for *Schistosoma mansoni* infection. Ova counts were not done for *Ancyclostoma duodenale* (hookworm) because of a delay in the examination of the Kato-Katz thick smears.

### Setting

Adama town is located to the east of Addis Ababa and has a total population of 244 435. In the town, most of the health institutions are privately owned. There are 12 private clinics, two public health centres and one private and one public hospital in the town.

### Design

A cross-sectional study was conducted to determine the prevalence of intestinal parasitic infections and malnutrition amongst first-cycle (Grade 1–4) primary school children in Adama town, eastern Ethiopia, from 19 December 2007 to 24 February 2008 (during the dry or winter climatic season). During the study period, there were 11 495, 4628, 1008 and 1371 children enrolled in government, private, public (charity-based) and non-governmental organisation (NGO) first-cycle elementary schools, respectively. A statistical formula for estimation of a single population proportion was used to estimate a sample size of 384.^10^ Due to non-response of some parents and/or guardians, a total of 358 schoolchildren were included in the study. Schools were stratified into government, private, public and NGO schools, after which one school was randomly selected from each stratum. Proportional allocation was done to determine the number of children from each school. Finally, a systemic random sampling technique using students’ rosters was applied to select the study subjects from each school.

### Analysing

The data were analysed using the EPINFO version 3.3 computer program. The association between intestinal parasitic infection and socio-economic factors was statistically tested using logistic regression analysis. The effect of these factors and helminthic infection on malnutrition was also tested.

## Results

The result of the stool examination by means of the formol- ether concentration technique showed that 127 (35.5%) children tested positive for one or more intestinal parasitic infection. The most frequent parasite identified was *Entamoeba histolytica/dispar* (12.6%), followed by *Hymenolopis nana* (8.9%) and *Giardia lamblia* (3.4%). The aggregate prevalence of protozoa (*E. histolytica/dispar* and *G. lamblia*) and helminths was nearly similar. Out of the total number of children investigated, 18.2% tested positive for different helminthic infections and 16% tested positive for cysts of *G. lamblia* and *E. histolytica/dispar. H. nana* was the most frequently encountered parasite amongst helminths (8.9%), whilst the least was *S. mansoni* (0.3%) (Table 1). The Kato- Katz smears showed that the majority of infections with *A. lumbricoides* and *T. trichiura* were light. Moderate intensities were found in a few of the children, but no children were found to be heavily infected (Table 2).

**TABLE 1:** Prevalence of parasitosis amongst schoolchildren.

Helminthic infections	% (*n* = 358)
*Taenia species*	2.00
*S. mansoni*	0.30
*G. lamblia*	3.40
*E. histolytica/dispar*	12.60
*T. trichiura*	1.10
*E. vermicularis*	0.60
*S. stercoralis*	0.60
*A. duodenale*	2.20
*A. lumbricoides*	2.50
*H. nana*	8.90

**Total**	**35.50**

*n*, number of schoolchildren.

**TABLE 2:** Intensity of helminthic infections.

Parasite	Intensity	Number of cases
*Ascaris lumbricoides*	Light	9
	Moderate	2
	Heavy	0
*Trichuris trichiura*	Light	3
	Moderate	1
	Heavy	0
*Schistosoma mansoni*	Light	0
	Moderate	1
	Heavy	0

The prevalence of intestinal parasitic infection amongst male children was 30.7% and 39.0% amongst female children, which had no significant difference (*p* > 0.05). The magnitude of parasitic infections was not dependent on the age of the study subjects, children's family income or educational status (*p* > 0.05). There was no significant difference of intestinal parasitic infection rate amongst schools (Table 3).

**TABLE 3:** Prevalence of intestinal parasitic infections amongst schoolchildren by sex, age, socio-economic factors and school type.

Prevalence classifications	Decription	Intestinal parasitic infection		*p*-value	OR	95% CI for OR

		Positive	Negative			
Sex	Male	30.0% (47/153)	69.3% (106/153)	0.164	0.727	0.464–1.139
	Female	39.0% (80/205)	61.0% (125/205)	-	-	-
Age group (in years)	6–9	34.4% (74/215)	65.6% (141/215)	0.148	0.442	0.146–1.337
	10–13	34.9% (45/129)	65.1% (84/129)	0.165	0.449	0.145–1.391
	14–17	42.9% (6/14)	57.1% (8/14)	-	-	-
Family income	< 200 ETB	39.8% (74/186)	60.2% (112/186)	0.382	0.732	0.365–1.471
	201–500 ETB	28.3% (28/99)	71.7% (71/99)	0.134	0.587	0.293–1.179
	> 500 ETB	34.2% (25/73)	65.8% (48/73)	-	-	-
Educational status of mother	Illiterate	40.0% (32/80)	60.0% (48/80)	0.616	1.232	0.688–3.464
	Elementary school	41.9% (57/136)	58.1% (79/136)	0.407	1.325	0.976–3.290
	Secondary school and above	26.8% (38/142)	73.2% (104/142)	-	-	-
Educational status of father	Illiterate	40.0% (32/80)	60.0% (48/80)	0.616	1.232	0.688–3.464
	Elementary school	41.9% (57/136)	58.1% (79/136)	0.407	1.325	0.976–3.290
	Secondary school and above	26.8% (38/142)	73.2% (104/142)	-	-	-
School type	Government	39.5% (90/228)	60.5% (138/228)	0.980	0.989	0.431–2.269
	Private	24.7% (18/73)	75.3% (55/73)	0.274	0.600	0.240–1.500
	Public	32.0% (8/25)	68.0% (17/25)	0.540	0.697	0.220–2.209
	NGO	34.4% (11/32)	65.6% (21/32)	-	-	-

OR, odds ratio; CI, confidence interval.

The overall prevalence of malnutrition was 21.2%. Out of the studied schoolchildren, 12.6%, 1.4% and 7.2% were stunted (Z-score of HA < 2SD), wasted (Z-score of WH < 2 and BMI < 18.5) and underweight (Z-score of WA < 2SD), respectively. The proportion of malnourished male children was higher than that of female children (*p* < 0.05; OR 2.214) (Table 4).

**TABLE 4:** Prevalence of malnutrition by age, sex and school type.

Prevalence classifications	Decription	Malnutrition		*p*-value	OR	95% CI for OR

		Yes	No			
Sex	Male	27.5% (42/153)	72.5% (111/153)	0.004	2.214	1.285–3.813
	Female	16.6% (34/205)	83.4% (171/205)	-	-	-
Age group	6–9	22.2% (39/176)	77.8% (137/176)	0.240	0.258	0.080–0.833
	10–13	24% (31/129)	76.0% (98/129)	0.085	0.356	0.110–1.155
	14–17	42.9% (6/14)	57.1% (8/14)	-	-	-
School type	Government	24.6% (56/228)	75.4% (172/228)	0.817	0.898	0.447–2.777
	Private	8.2% (6/73)	91.8% (67/73)	0.06	0.317	0.096–1.051
	Public	28.0% (7/25)	72.0% (18/25)	0.999	1.001	0.284–3.527
	NGO	9.2% (7/32)	78.1% (2532)	-	-	-

OR, odds ratio; CI, confidence interval; NGO, non-governmental organisation.

The logistic regression analysis of socio-economic factors showed that those children whose family income was less than 200 and between 200 and 500 EBR were more affected by malnutrition (*p* < 0.008). However, the prevalence of malnutrition was not affected by parental educational status, ethnicity and religion (Table 5). Out of the total positive children with different intestinal infections, 22.4% were malnourished, but the observed difference in nutritional status between infected and non-infected children was not statistically significant (Table 6).

**TABLE 5:** Prevalence of malnutrition amongst schoolchildren by socio-economic factors.

Prevalence classifications	Decription	Malnutrition		*p*-value	OR	95% CI for OR

		Yes	No			
Family income	< 200 ETB	32.3% (60/186)	67.7% (126/186)	0.002	9.297 9.297	6.810–17.109
	201–500 ETB	15.2% (15/99)	84.8% (84/99)	0.008	6.558	2.104–13.280
	>500 ETB	1.4% (1/73)	98.6% (72/73)	-	-	-
Educational status of mother	Illiterate	30.5% (36/118)	69.5% (82/118)	0.92	0.951	0.358–2.530
	Elementary school	28.9% (22/132)	83.3% (110/132)	0.244	0.951	0.252–1.395
	Secondary school and above	16.7% (18/108)	83.3% (90/108)	-	-	-
	Illiterate	31.3% (25/80)	68.8% (55/80)	0.477	0.705	0.269–1.849
Educational status of father	Elementary school	18.4% (25/136)	81.6% (111/136)	0.477	0.49	0.223–1.075
	Secondary school and above	18.3% (26/142)	81.7% (116/142)	-	-	-
	Oromo	24.4% (39/160)	75.6% (121/160)	0.32	2.027	0.504–8.141
Ethnicity	Amhara	17.4% (21/121)	82.6% (100/121)	0.695	1.346	0.305–5.952
	Tigre	30% (9/30)	70.0% (21/30)	0.295	2.433	0.461–12.837
	Gurage	12.5% (3/24)	87.5% (21/24)	0.837	0.831	0.142–4.866
	Others	17.4% (4/23)	82.6% (19/23)	-	-	-
Religion	Orthodox	23.4% (58/248)	76.6% (190/248)	0.309	0.115	0.002–7.432
	Muslim	22.8% (13//57)	77.2% (44/57)	0.339	0.128	0.002–8.687
	Protestant	8.0% (4/50)	92.0% (46/50)	0.111	0.03	0.000–2.230
	Others	33.3% (1/3)	66.7% (2/3)	-	-	-

ETB, Ethiopian birr; OR, odds ratio; CI, confidence interval.

**TABLE 6:** Helminthic infection and malnutrition amongst schoolchildren.

Malnutrition	Helminthic infection			*p*-value	OR	95% CI for OR

	Yes	No	Total			
Yes	17 (22.4%)	59 (77.6%)	76	0.285	1405	0.754–2.618
No	48 (17%)	234 (83%)	282	-	-	-

**Total**	**65**	**293**	**358**	**-**	**-**	**-**

OR, odds ratio; CI, confidence interval.

## Discussion

The overall prevalence of intestinal parasitic infections amongst the schoolchildren was 35.5%, which is low compared to different studies conducted in Mexico (67%), Nigeria (54.7%) and Ethiopia (68.4% and 83.8%).^3, 4, 7, 8^ The most frequently found parasite in this study was *E. histolytica/dispar* (12.6%), followed by *H. nana* (8.9%) and *G. lamblia* (3.4%). The prevalence of *E. histolytica/dispar* (12.6%) reported in this study is comparable to the 12.7% from another study in Ethiopia.^7^ The higher prevalence of this parasite compared to helminths might be due to the fact that cysts are more resistant to dry weather than eggs of helminths.^1^

The low prevalence (2.5%) of geo-helminths, *A. lumbricoids*, is attributable to the fact that geo-helminths require hot and humid weather and wet soil.^1^ Because the study area is lowland lying in the rift valley with dry weather and soil, environmental factors can affect the survival of the ova of these parasites in the external environment so that transmission can be hindered. In agreement with this study, a study conducted in lowlands of Ethiopia has also shown a low prevalence of *A. lumbricoides* in children (1.5%).^15^

*H. nana* was found to be the most prevalent (8.9%) parasite comparable to the report from the study conducted in Babile town, eastern Ethiopia,^16^ but it was higher than that of the study in Mexico.^3^ The high rate of *H. nana* observed in this study indicates that hygienic practices of the schoolchildren in the study area are poor and that it is an important factor for auto- infection and transmission to others.^1^ Unlike geo-helminths, this parasite does not require the external environment for maturation of eggs, which means the lifecycle of this parasite cannot be affected. The high prevalence might therefore be due to its high transmission.

The prevalence of malnutrition was relatively higher in public schools than in the rest of the schools, even though the difference was not significant (p > 0.05). The overall prevalence of malnutrition in this study was 21.2% and the most frequent type of malnutrition was stunting (12.6%), which was in agreement with a finding from the study conducted in Babile town.^16^ In agreement with other studies,^5, 7, 10^ stunting was the leading type of malnutrition observed in this study. Stunting is associated with chronic conditions such as prolonged food shortage.^1^ Therefore, the observed result in the present study could be due to a prolonged shortage of balanced meals, especially amongst children from poor families.

## Conclusion

The observed prevalence of *E. histolytica/dispar* and *H. nana* is of public health importance and requires control measures. In addition, the magnitude of malnutrition amongst children from poor families also warrants intervention strategies. The prevalence of malnutrition was not associated with helminthic infection and the observed stunting may be due to a prolonged shortage of a balanced diet. Therefore, prevention and control measures can focus on regular school health education programmes on the prevention of intestinal parasitic infections, school health programmes for the assessment of malnutrition and health education for parents and/or guardians on how to prevent intestinal parasitic infection and malnutrition, as well as the provision of one subsidised school meal per child per day.
